# Integrative Genomic and AI Approaches to Lung Cancer and Implications for Disease Prevention in Former Smokers

**DOI:** 10.3390/ijms27010521

**Published:** 2026-01-04

**Authors:** Katya H. Bénard, Vanessa G. P. Souza, Greg L. Stewart, Katey S. S. Enfield, Wan L. Lam

**Affiliations:** 1 British Columbia Cancer Research Institute, Vancouver, BC V5Z 1L3, Canada; 2 Interdisciplinary Oncology Program, University of British Columbia, Vancouver, BC V6T 1Z4, Canada; 3 Department of Pathology and Laboratory Medicine, University of British Columbia, Vancouver, BC V6T 1Z7, Canada

**Keywords:** smoking cessation, genomic profiling, persistent molecular alterations, lung cancer, DNA methylation, artificial intelligence, precision prevention, field cancerization

## Abstract

Tobacco smoking accounts for nearly 90% of lung cancer deaths worldwide, yet the mechanisms underlying persistent cancer risk in former smokers are not fully understood. Epidemiological evidence shows that more than 40% of lung cancers develop over 15 years after cessation, demonstrating that while some smoking-induced molecular alterations resolve rapidly, others remain as long-lasting scars that promote carcinogenesis. This review synthesizes longitudinal and cross-sectional genomic, epigenomic, and transcriptomic studies of airway and lung tissues to distinguish persistent from nonpersistent smoking-induced molecular alterations. Persistent alterations include somatic mutations in *TP53* and *KRAS*, DNA methylation at tumor suppressor loci, dysregulated noncoding RNAs, chromosomal instability, and epigenetic age acceleration. Nonpersistent changes, such as acute inflammatory responses and detoxification pathways, generally normalize within months to several years following cessation. Multi-omics profiling reveals coordinated patterns of dysregulation consistent with field cancerization in former smokers. In addition, the integration of multi-omics data with artificial intelligence may enable composite molecular signatures for stratifying high-risk former smokers, link molecular persistence to clinical outcomes, and inform chemoprevention strategies. Collectively, these observations clarify which molecular alterations sustain long-term cancer risk despite smoking cessation and highlight opportunities for precision prevention and earlier detection in high-risk populations.

## 1. Introduction

Lung cancer remains the leading cause of cancer death worldwide, accounting for approximately 1.8 million deaths annually and representing 18% of all cancer deaths [1]. Despite progress in early detection and treatment, mortality rates remain high due to late-stage diagnoses, persistent global smoking prevalence, limited specificity and sensitivity of screening methods, and treatment resistance. Non-small-cell lung cancer (NSCLC) comprises 87% of lung cancer cases, with lung adenocarcinoma (LUAD) and lung squamous cell carcinoma (LUSC) as the primary histologic subtypes [2]. Small-cell lung cancer (SCLC) accounts for the remaining 13% of cases [2]. Among lung cancers, LUSC and SCLC have the strongest association with tobacco exposure, with more than 95% of SCLC cases occurring in individuals with a history of tobacco use [3,4]. Smoking is the predominant risk factor for lung cancer and is responsible for approximately 85% of cases globally [5]. Risk increases with both duration and quantity of smoking [2]. Although global tobacco use has declined from 32.7% of adults in 2000 to 21.7% in 2020, with projections to fall below 20% by 2025, former smokers continue to face persistently elevated risk of lung cancer that can last for decades after quitting [6,7,8]. This enduring susceptibility, combined with the large population of individuals with a history of smoking, represents a substantial and ongoing health burden from tobacco-related lung cancers.

Tobacco smoke induces cytotoxic damage to airway epithelial cells, resulting in oxidative stress, DNA damage, and chronic inflammation, which are key factors that promote smoking-related lung disease [9,10]. Chronic exposure to tobacco carcinogens leads to the formation of DNA adducts, resulting in oncogenic mutations in critical genes, including *TP53* and *KRAS* [11,12,13]. Tobacco smoke is also associated with aberrant DNA methylation of promoter regions in tumor suppressor genes such as *CDKN2A*/p16, silencing their expression [14,15]. These processes promote basal cell hyperplasia and squamous metaplasia in the airway epithelium, contributing to epithelial remodeling, barrier dysfunction, and other early histopathologic changes that represent or precede premalignant lesions [16,17]. For example, polycyclic aromatic hydrocarbons (PAHs), compounds that primarily act as local carcinogens in the bronchial epithelium, form PAH-DNA adducts that generate characteristic mutations such as the excess G to T transversions that are commonly observed in smoking-related squamous tumors [15]. These PAH-associated mutational patterns, including site-specific damage at *TP53* hotspots, are consistent with genomic alterations underlying LUSC arising from chronically smoke-exposed airway epithelium. The tobacco-specific nitrosamine 4-(methylnitrosamino)-1-(3-pyridyl)-1-butanone (NNK) acts as a systemic lung carcinogen that mainly induces adenocarcinoma in experimental models. This finding is consistent with the prevalence of LUAD among smokers [15]. The accumulation of these molecular insults across the respiratory tract creates a “field cancerization” effect in which large areas of cells harbor pre-neoplastic changes [18,19,20]. Epigenetic studies have reported that tobacco smoke is associated with reproducible alterations in DNA methylation and dysregulation of miRNAs. Histone modifications associated with tobacco smoke exposure are also well documented [21].

Not all molecular changes revert after smoking cessation. Some remain after decades of abstinence and are therefore described as “persistent” or “irreversible.” Other molecular alterations gradually normalize to levels of never-smokers and are referred to as “reversible” or “nonpersistent” [17,22,23]. These terms are not defined with uniform temporal thresholds across studies. Follow-up durations vary, ranging from months to several years, and in some instances, more than a decade after smoking cessation. Therefore, persistence is more appropriately viewed as a spectrum rather than a fixed, universally applicable cutoff, reflecting differences in molecular class, genomic context, tissue type, and study design. In this review, these alterations are referred to as persistent when they remain significantly altered in long-term former smokers or are reproducibly observed across independent cohorts and tissue types, even when assessed years after cessation. Nonpersistent alterations show partial or complete reversion toward never-smoker levels in longitudinal or cross-sectional cessation studies, typically within months or several years. This framework emphasizes reproducibility and signal durability rather than a fixed cessation interval. Certain features, such as DNA methylation marks, reduced DNA repair capacity, and self-sustaining signaling loops, may persist long after smoke exposure ends and may contribute to elevated long-term cancer risk despite cessation. Epidemiological data demonstrate that excess cancer risk declines slowly after cessation, plateauing at about 20% above baseline only 20 years after quitting [7].

Persistent changes can be leveraged for risk stratification and allow opportunities for early detection strategies and personalized surveillance among high-risk former smokers. Identifying nonpersistent alterations may reveal reversible molecular targets for chemoprevention, allowing intervention before persistent changes establish malignancy. Early detection is especially important in lung cancer, as most cases are detected in advanced stages, when curative options are limited [24,25].

Distinguishing between persistent and nonpersistent molecular changes is critical for precision prevention. However, these alterations span multiple molecular classes (e.g., genetic, epigenetic, transcriptomic, proteomic, metabolomic), exhibit high inter-patient variability, and are captured in vast, complex datasets that are difficult to interpret using conventional analytic approaches [17,21]. Cohorts are often relatively small and diverse and have limited labeling of persistence, which is typically determined by longitudinal assessments. The process of molecular recovery also takes time, making it difficult for current methods to capture risk trajectories. In addition, models created from one population frequently fail to generalize to others. Advanced computational methods, including artificial intelligence (AI) and machine learning (ML), are emerging as valuable approaches for integrating multi-omics data, distinguishing short-lived from enduring molecular scars, and translating the findings into actionable biomarkers for monitoring and intervention [26,27,28].

Although extensive research has characterized smoking-related molecular alterations and AI-based cancer risk prediction has advanced substantially, an important gap remains in how these areas are integrated to distinguish persistent from nonpersistent changes and to inform prevention strategies in former smokers. To address this gap, relevant literature was identified through targeted searches of biomedical databases using terms related to smoking cessation, persistent versus nonpersistent molecular alterations, airway and lung epithelium, field cancerization, and longitudinal or cross-sectional study designs. Studies were prioritized based on relevance to persistence dynamics, tissue context, methodological rigor, and reproducibility across independent cohorts. This review examines the molecular landscape of persistent and nonpersistent alterations induced by tobacco exposure, discusses implications for clinical risk reduction strategies, and appraises the potential and limitations of AI-based approaches in advancing precision prevention and personalized approaches to lung cancer control.

## 2. Smoking-Induced Molecular Changes

Tobacco smoke is a complex mixture of toxic constituents, containing over 7000 chemical compounds, at least 69–80 of which are recognized carcinogens by the International Agency for Research on Cancer (IARC) and U.S. National Cancer Institute [15,29,30,31,32]. These include PAHs, tobacco-specific nitrosamines, heavy metals like arsenic and cadmium, and radioactive elements such as polonium-210 [33]. Components of combustible cigarette smoke also include aromatic amines, reactive aldehydes, and benzene, which collectively contribute to DNA damage, oxidative stress, and genomic instability [15]. Nicotine and certain nitrosamines can aberrantly activate cell signaling pathways that act as tumor-promoting rather than directly mutagenic agents, promoting the survival and clonal expansion of damaged epithelial cells [15,18]. These mechanisms reflect exposure to combustion-derived toxicants in cigarette smoke, and therefore, the molecular alterations discussed in this review should not be extrapolated to non-combustible nicotine delivery systems, for which long-term genomic and cancer risk data remain limited. Despite these well-characterized carcinogenic mechanisms, the temporal dynamics of smoking-induced molecular alterations present challenges for risk assessment and intervention.

The clinical consequences of these carcinogenic mechanisms are evident in persistent cancer risk following cessation. Even after 25 years of cessation, lung cancer risk in former smokers remains over three times higher than in never-smokers [34,35]. Analysis of the Framingham Heart Study, encompassing the Original (*n* = 3905) and Offspring (*n* = 5002) cohorts with longitudinal follow-up for smoking exposure and lung cancer incidence from 1954 to 2013, found that 40.8% of lung cancers in former smokers occurred after more than 15 years since quitting, demonstrating the long temporal window where molecular alterations continue to affect cancer risk [35]. Although quitting at any age reduces risk, earlier cessation greatly improves outcomes, as quitting before age 40 lowers the risk of death from tobacco-related disease by ~90% [34]. Cessation at ages 60, 50, 40, or 30 years extends life expectancy by approximately 3, 6, 9, or 10 years, respectively, with cumulative lung cancer incidence declining progressively with longer cessation periods [36].

The persistence of smoking-induced molecular changes is contingent on the nature of the alteration, whether epigenetic, transcriptional, or genetic, and on the genomic loci involved [17,37,38]. While many expression changes normalize after cessation, aberrant DNA methylation at regulatory sites may persist for decades [37,38]. Persistence of smoking-related epigenetic changes appears to be site-specific and not directly dependent on exposure intensity or duration, with certain loci remaining altered for decades after cessation [37,38]. However, cumulative lifetime exposure has been associated with accelerated epigenetic aging, reflecting the broader effects of long-term cigarette smoke exposure [39]. Genetic variation and cigarette smoking independently influence DNA methylation through primarily distinct sets of loci. This differential impact may contribute to interindividual differences in susceptibility to smoking-related disease [40,41]. Collectively, these accumulated epigenetic and genetic alterations in the bronchial epithelium may represent early molecular events in the pathogenesis of smoking-related lung cancers [42,43].

The sensitivity of airway epithelium to tobacco exposure shows a dose-response relationship with no clear threshold for molecular alterations. Low levels of exposure (0.1 ± 0.3 pack-years) produce detectable transcriptomic changes in small airway epithelium, with 34% of differentially expressed genes observed between never-smokers and low-level exposed individuals [44]. The most sensitive genes to tobacco metabolites include *PLA2G10* and *CXCL6*, which respond to nicotine urine levels below 2 ng/mL. Cotinine-responsive genes, such as *CYP2E1* and *GAD1*, show alterations at urine concentrations of approximately 6.2–7.3 ng/mL, demonstrating that molecular damage begins with minimal exposure [44].

### 2.1. Nonpersistent Molecular Changes

#### 2.1.1. Transcriptomic and Functional Recovery

Many smoking-induced changes are nonpersistent, particularly those in xenobiotic metabolism and acute stress pathways. A core set of nine genes (*CYP1B1*, *ALDH3A1*, *AKR1B10*, *AKR1C1*, *AKR1C2*, *AKR1C3*, *MUC5AC*, *NQO1*, and *SCGB1A1*), identified through a cross-sectional comparison of bronchial epithelium in current and former smokers, consistently return to normal expression levels [45]. Longitudinal studies found that genes related to xenobiotic metabolism (*CYP1A1*, *CYP1B1*, *ALDH3A1*) and homeostasis (*MUC2*, *MUC13*) in the nasal epithelium are among the most rapidly reversible, with expression levels reverting toward baseline within 4 weeks after cessation [46]. 88.2% of smoking-upregulated gene expression changes showed downregulation by 8 weeks, with 11.8% beginning to decrease within 4 weeks, indicating an early reversal trend following smoking cessation [46]. The earliest molecular responses to smoking cessation include rapid epigenetic recovery within months, characterized by widespread changes in DNA methylation at CpG sites and alterations in cellular stress and metabolic pathways. Short-term cessation studies (3–6 months) reveal global decreases in DNA methylation affecting 3878 CpG sites, with 694 sites showing increased methylation and 3,184 showing decreased methylation [47]. These methylation changes correlate with improved lung function and reduced inflammatory biomarkers, indicating that molecular recovery begins immediately upon cessation.

Inflammation and stress response genes also demonstrate recovery. Examples include *MMP10* in human airway epithelial cells and cytokines such as IL-1α, TNF-α, CCL2, and CCL3, which normalize in animal studies, alongside immune cell counts [22,48]. Broader metabolic recovery takes longer, as metabolic and antioxidant expression profiles of former smokers resemble never-smokers after ~2 years [22]. Genes involved in nucleotide metabolism, xenobiotic metabolism, and mucus secretion (e.g., *TFF3*, *CABYR*, *ENTPD8*) recover, with partial reversal of *MUC5AC* [49]. The PI3K pathway, an early smoke-responsive signaling axis, has also been found to normalize following months of cessation, enhanced by targeted intervention with myo-inositol treatment [50].

#### 2.1.2. Epigenetic and microRNA Recovery

While most studies focus on airway epithelial changes, systemic effects are also evident in blood-derived markers, demonstrating broader epigenetic recovery after smoking cessation. Although many methylation scars are persistent, a larger fraction revert to baseline levels. Analysis of whole-blood DNA methylation data identified 602 nonpersistent versus 149 persistently differentially methylated CpG sites [38]. Time-dependent reversal patterns of CpGs have also been observed: 32 CpG sites showed significant change within 4 years of cessation and 30 within 5–14 years (10 sites shared between these groups), with only *AHRR* cg26703534 persisting after 14 years [37]. Key sentinel sites, including *AHRR* and *F2RL3*, demonstrate robust reversion toward never-smoker levels in long-term blood-based studies [51]. Circulating gene expression biomarkers complement tissue-based findings, with blood-based analysis identifying 94 nonpersistent genes that normalize to never-smoker levels and 31 genes that revert more slowly out of the 132 smoking-related genes analyzed [52]. Similarly, ~65% of smoking-altered miRNAs in small airway epithelium returned to baseline within 3 months of quitting smoking [53]. Strulovici-Barel et al. reported that 67% of smoking-dysregulated genes reversed within 12 months, while persistent apoptosis and growth-related genes were more resistant [17]. These findings demonstrate that while many molecular alterations regress after cessation, a subset of alterations persist and likely sustain risk. Blood-based and airway-based biomarkers may serve as non-invasive tools for the surveillance of cessation success and long-term molecular damage, with potential applications in population-level risk assessment and screening.

### 2.2. Persistent Molecular Changes

**Figure 1:**
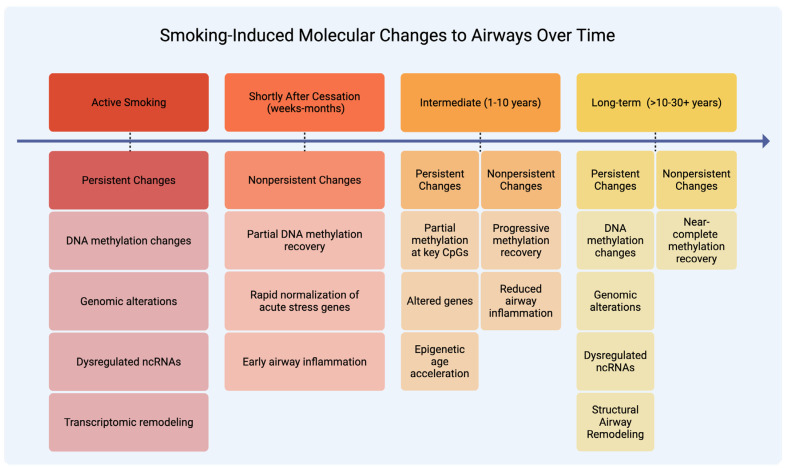
Smoking-Induced Molecular Changes to Airways Over Time. This figure depicts how active smoking and cessation affect airway molecular and cellular changes over time. The red column indicates active smoking, dark orange represents the period shortly after cessation (weeks-months), light orange shows the intermediate period after cessation (1–10 years), and yellow indicates the long-term period after cessation (>10–30+ years). Within each time period, the segments are divided into persistent and nonpersistent changes where applicable. Shortly after smoking cessation, some changes show rapid or partial recovery (nonpersistent), such as the normalization of acute stress gene expression and reduced airway inflammation. Other changes remain for years to decades (persistent), including DNA methylation abnormalities at key CpGs, genomic scars, dysregulated ncRNAs, epigenetic age acceleration, and structural airway remodeling. Figure 1 illustrates how persistent molecular alterations leave lasting molecular marks that contribute to lung cancer risk long after exposure ends.

Smoking leaves behind a range of persistent molecular changes that continue to influence airway biology long after cessation. These include irreversible DNA mutations, sustained shifts in gene expression and regulation, epigenetic reprogramming, and immune or structural remodeling, each described in the subsections below. Figure 1 outlines the timeline of these processes, highlighting nonpersistent changes that recover within months to years versus persistent changes that remain for decades.

#### 2.2.1. Genetic Alterations

Structural genetic lesions represent the most permanent consequences of smoking. Studies have found that about 62% of former smokers, with an average cessation period of 27 months, harbor clonal genetic alterations in histologically normal lung tissue [43]. These include loss of heterozygosity at 3p14 (*FHIT*, observed in 75% of informative smokers overall), 9p21 (*CDKN2A*, 57%), and 17p13 (*TP53*, 18%). Among former smokers, LOH at 3p14 was detected in 45%, compared with 88% of current smokers (*p* = 0.01).

Unlike some partially reversible epigenetic and transcriptomic changes, DNA lesions are fundamentally irreversible. Once the mutations occur, they last for the lifetime of that cell. Whole-genome sequencing of 632 single-cell-derived bronchial epithelial colonies from current, former, and never-smokers shows that tobacco exposure adds thousands to tens of thousands of mutations per cell, and that these alterations persist in affected cell lineages [54]. The clonal patches harboring these mutations remain as permanent genomic scars in former smokers.

#### 2.2.2. Gene Expression and Regulatory Changes 

Longitudinal small airway epithelium studies show that a subset of smoking-dysregulated genes remains abnormally expressed after cessation. In one 12-month study, 53 (11%) of 475 genes did not normalize, including *CYP1B1*, *PIR*, *ME1*, *TRIM16*, with apoptosis and proliferation pathways most resistant [17]. Spira et al. identified 13 persistently altered genes detectable even 20–30 years post-cessation [22]. These included decreased expression of potential tumor suppressor genes such as *TU3A* and *CX3CL1*, and increased expression of the oncogenes *HN1* and *CEACAM6*. In addition, three metallothionein genes located at 16q13 remained persistently downregulated, suggesting a fragile site for DNA damage in smokers. Beane et al. identified 28 persistently dysregulated genes in large airway epithelium [23]. The persistent down-regulation of genes such as *SULF1*, *UPK1B*, and metallothioneins suggest the clonal selection of altered epithelial cells that maintain smoke-induced molecular changes. MiRNAs also contribute to persistent remodeling. Of 34 small airway epithelium miRNAs altered by smoking, 12 remained dysregulated after 3 months of cessation, including miR-218, miR-133a/b, miR-487b, and miR-1246 [53]. The target genes of these miRNAs are primarily enriched for the Wnt/β-catenin signaling pathway. In the airway epithelium of current smokers, a self-amplifying *EGFR*–amphiregulin autocrine loop was identified that is absent in never-smokers and drives basal-cell hyperplasia and squamous metaplasia [55]. This smoke-induced feedback maintains *EGFR* activation and may contribute to persistent epithelial remodeling and increased susceptibility to smoking-related lung disease. Distinct miRNA expression patterns in LUAD based on smoking history include 66 miRNAs showing differential alterations: 25 in current smokers, 14 in former smokers, and 27 in never-smokers [56]. These smoking status-specific miRNA networks show prognostic significance and suggest that the molecular impact of smoking influences treatment response and survival outcomes. Tissue-based analysis identified six distinct persistently dysregulated genes—*LEF1*, *ADAMTS1*, *SFXN1*, *CST7*, *CCR7*, and *GNB2L1*—as markers of lasting gene expression changes in former smokers, highlighting differences in biomarker signatures associated with smoking cessation [52].

#### 2.2.3. Epigenetic Modifications

Tobacco smoke induces long-lasting epigenetic alterations, with DNA methylation changes persisting for decades after cessation and influencing key regulatory pathways across the genome. Epigenome-wide association studies using whole-blood DNA have identified 149 CpG sites that remain differentially methylated >35 years post-cessation [38]. Key smoking-associated methylation sites include cg05575921 in the *AHRR* gene, methylation changes in *F2RL3* and *GFI1*, and broader differentially methylated regions such as 6p21.33 on chromosome 6 and 2q37.1 on chromosome 2 [38]. Genome-wide methylation analysis has confirmed the extensive epigenetic impact of smoking across multiple loci, with 972 CpG sites showing significant methylation differences (>5%), and 187 of these CpG sites were replicated in an additional cohort [57]. The sentinel site cg05575921 in *AHRR* demonstrates the highest level of detectable DNA methylation changes, with ~24% hypomethylation in current smokers. The widespread nature of these changes, detected across all autosomes in whole blood, includes altered protein binding at the sentinel site cg05575921 in *AHRR*, suggesting potential effects on transcription factor binding and gene expression regulation. These findings demonstrate that smoking induces broad epigenetic remodeling in blood-derived immune cells and that these alterations may extend beyond traditional cancer-associated genes [57]. Single-cell methylation profiling of bronchial basal progenitors isolated via bronchial brushing reveals persistent genome-wide hypomethylation affecting loci such as *KRAS*, *ROS1*, *CDKN1A*, *CHRNB4*, *CADM1* [42]. Persistent marks also overlap age-associated CpGs and Polycomb targets, implicating developmental and immune-related pathways consistent with aging-associated epigenetic remodeling [58].

While most persistent alterations are characterized in airway and lung tissue, blood-based profiling may be a less invasive method of assessing the systemic and long-term molecular impacts of smoking. The functional consequences of persistent methylation changes have been demonstrated in prospective cohort studies that examine pre-diagnostic blood samples. Analysis of 796 case-control pairs throughout four independent cohorts showed that hypomethylation at *AHRR* cg05575921 and *F2RL3* cg03636183 was highly associated with future lung cancer risk, with odds ratios of 0.37 (95% CI: 0.31–0.54) and 0.40 (95% CI: 0.31–0.56) per standard deviation increase in methylation, respectively [59]. These associations remain strong after adjusting for smoking status, indicating that methylation alterations may have independent predictive value outside of smoking history alone. Mediation analysis of methylation at these two specific CpG sites in *AHRR* and *F2RL3* estimated that approximately 37% (95% CI: 19–66%) of the total effect of tobacco smoking on lung cancer odds is mediated by methylation at these loci [59]. This suggests that these epigenetic changes may play a causal role rather than serving as exposure biomarkers alone. The authors note that this observation could partly reflect chance or residual confounding, warranting cautious interpretation. On average, lung cancer cases were diagnosed about 3.88–9.6 years after blood collection in NOWAC, MCCS, and NSHDS cohorts, exemplifying the long-term predictive capacity of the persistent methylation changes [59]. Cross-sectional analysis of former smokers at differing time points post-cessation shows that *AHRR* and *F2RL3* methylation levels gradually approach never-smoker levels. The most substantial recovery took place within the first 10 years after quitting, although complete normalization was not achieved in even long-term former smokers.

Epigenetic age acceleration is another component of persistent smoking-induced alterations. Smoking has been found to increase the epigenetic age of airway cells by an average of 4.9 years and of lung tissue by 4.3 years [60]. After cessation, epigenetic age acceleration reversed in airway cells to never-smoker levels, but not in lung tissue. This incomplete reversal suggests that long-lived or slowly renewing cells in the lung retain smoking-induced molecular damage, potentially maintaining a pro-oncogenic tissue environment even after cessation. The clinical relevance of epigenetic changes is further supported by airway-specific methylation patterns in the lung. Former smokers, who had quit at least two years before study inclusion, displayed chronic mucus hypersecretion and increased promoter methylation of lung cancer risk genes, such as *SULF2*, when compared to asymptomatic former smokers [61]. Therefore, persistent respiratory symptoms may be good indicators of lasting epigenetic dysregulation, even after cessation.

#### 2.2.4. Immune and Structural Consequences

Persistent immune dysfunction includes dysregulation of neutrophil-mediated immunity and interferon-γ-related pathways, contributing to elevated lung cancer risk lasting over 10 years after smoking cessation [62]. Animal models confirm structural irreversibility, as elevated IL-12, reduced IL-10, alveolar enlargement, right ventricular hypertrophy, and ongoing inflammation persisted 8 weeks after smoke exposure ended in A/J mice [48]. In longer-term mouse models, neutrophilic inflammation, macrophage accumulation, and destructive changes consistent with lung remodeling persisted six months after smoking cessation [63]. Chronic smoke-exposed mice also showed progressive alveolar damage and inflammation that lasted longer than exposure periods, suggesting ongoing structural deterioration after cessation [63]. In humans, adaptive immune changes appear more persistent than innate alterations. While innate responses normalize, cytokine response patterns from T cells stay altered in former smokers, potentially linked to epigenetic memory [64].

## 3. Role of AI in Advancing Strategies for Prevention and Intervention

AI has rapidly evolved from a niche computational tool into a widely used approach across science and medicine. Over the past decade, continuous advances in algorithms, computing power, and data accessibility have driven rapid growth in AI capabilities and applications. In oncology, AI has shown promising proof-of-concept success in improving early cancer detection, predicting treatment responses, and identifying molecular or imaging-based biomarkers, offering potential to accelerate research and support clinical decision-making [26,27,65,66].

ML and deep learning (DL) models provide computational frameworks to capture complex biological signals beyond single-gene markers [27,65]. Rather than relying on a handful of marker genes, these AI-driven approaches analyze integrated multi-omics datasets, including transcriptomic, epigenomic, proteomic, and metabolomic profiles, to identify composite molecular signatures that reflect coordinated changes across numerous features [67,68]. Such signatures may strengthen the ability to distinguish persistent from nonpersistent smoking-induced alterations. These methods aim to improve interpretability and generalizability by modeling coordinated biological signals rather than isolated features [26]. By applying these approaches to smoking-related airway and lung datasets, AI may support early detection by identifying individuals at elevated risk, enabling personalized risk stratification in former smokers, and revealing nonpersistent molecular pathways that could be targeted with early interventions [8,16,65]. By doing so, AI offers an outline for operationalizing the persistent and nonpersistent framework to improve prevention, monitoring, and treatment strategies [20,24,26].

Within the context of tobacco-associated lung carcinogenesis, one major challenge lies in distinguishing persistent from nonpersistent molecular alterations induced by smoking exposure [17,23,42,54]. This distinction is essential for understanding why some molecular changes revert after smoking cessation while others persist as durable molecular alterations that sustain cancer risk, and for developing effective, personalized prevention and intervention strategies [8,16]. However, efforts to characterize persistent and nonpersistent alterations generate extensive and complex datasets that are difficult to interpret with conventional analytical methods [26]. Current approaches are limited by fragmented multi-omics signals that lack integration across datasets, heterogeneous cohorts, incomplete labeling of persistence (generally labeled via longitudinal data showing temporal stability of alterations), and the barriers of modeling molecular recovery as a dynamic process [23,37,40,69]. The interplay of genetic susceptibility, cumulative exposure, and time since cessation further complicates the identification of truly causal, persistent alterations within a landscape of reversible changes [8,35,54]. 

**Figure 2:**
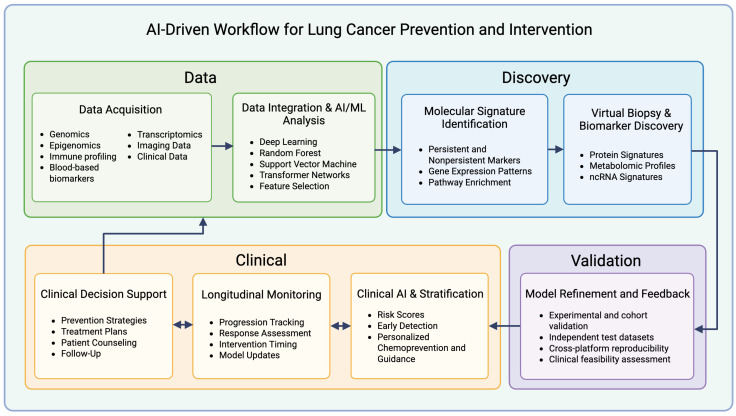
AI-Driven Workflow for Lung Cancer Prevention and Intervention. This schematic illustrates an eight-step workflow linking multi-source data acquisition, AI/ML analysis, molecular signature identification, validation and model refinement, and virtual biomarker discovery. Data inputs span genomics, epigenomics, transcriptomics, imaging, immune profiling, and blood-based biomarkers. Clinical insights from these analyses support risk stratification, personalized prevention, and treatment planning. Validated discovery outputs transition into clinical application, while longitudinal monitoring and bidirectional feedback between data integration, validation, monitoring, and decision support enable continuous model refinement and improved clinical relevance, highlighting approaches to distinguish persistent from reversible tobacco-related molecular changes in lung cancer.

To address these challenges, AI-driven approaches can be organized into a structured workflow that links molecular data with clinical application. Figure 2 provides an overview of this framework, beginning with data integration and multi-omics analysis, progressing through molecular signature identification, validation and model refinement, and extending into clinical decision support. Table 1 complements this figure by summarizing representative datasets, sequencing platforms, AI models, and software tools corresponding to each stage [70,71,72,73,74,75,76,77,78,79,80,81,82,83,84,85,86,87,88,89,90,91,92,93,94,95,96,97,98,99,100,101,102,103,104,105,106,107,108,109,110,111,112,113,114,115,116,117,118,119,120,121,122]. The example tools and models listed in Table 1 represent commonly used approaches across lung cancer risk prediction and multimodal analysis; persistence-specific applications remain comparatively limited and are discussed further in Section 4. Together, Table 1 and Figure 2 illustrate how AI may serve as a bridge between complex biological data and actionable strategies for lung-cancer prevention and intervention.

### 3.1. Identifying Molecular Signatures

AI-based ML and DL models are increasingly used to identify novel biomarkers and analyze complex biological datasets by leveraging coordinated changes across omics patterns rather than relying on single markers [27,123]. DL performs well at discerning complicated patterns in large datasets, which is optimal for the interpretation of smoking-induced molecular heterogeneity and for cohorts with incomplete or indirect labeling of persistence [26,124]. While the direct application of these models to differentiate between persistent and nonpersistent smoking-induced changes remains an emerging area of research, previous foundational work in oncology demonstrates the feasibility of this approach [125,126].

An important capability of AI is the ability to reclassify tumors with ambiguous features into well-defined molecular categories. For example, a DL model was successfully able to reclassify combined hepatocellular-cholangiocarcinoma (cHCC-CCA), which is a rare and biphenotypic cancer, into more distinct hepatocellular carcinoma (HCC) or intrahepatic cholangiocarcinoma (ICCA) categories [126]. This morphological reclassification was validated by its strong correlation with distinct spatial gene expression profiles and genetic alterations (e.g., TERT, CTNNB1, FGFR2) establishing that AI can connect histological patterns with functionally distinct molecular states [126]. This application may provide conceptual precedent for distinguishing persistent, high-risk molecular states from nonpersistent states in the airways of former smokers.

AI has also been applied to identify subtle but functionally important genetic alterations within broad genomic patterns [27,125]. For example, the DL tool “Dig” maps genome-wide somatic mutation rates and identifies driver mutations under positive selection by comparing the observed mutation counts to predicted neutral rates [125]. This tool has been used to reveal significant mutations in splice sites and 5’ untranslated regions that are linked to altered gene expression and frequently overlooked by traditional methods. This research sets a precedent for using AI to uncover specific, functionally significant and persistent mutations within a landscape of neutral or transient variation [125].

Together, these advances demonstrate that AI can identify biomarkers based on complex omics signatures that reflect coordinated alterations across molecular features. By extension, an adequately trained deep neural network could help elucidate the molecular signatures that differentiate persistent from nonpersistent changes in the airway epithelium of smokers. Numerous ML algorithms, such as Support Vector Machines (SVM), Random Forests (RF), and the Least Absolute Shrinkage and Selection Operator (LASSO), are often used for feature selection and classification, enabling the identification of salient molecular alterations from large-scale datasets [123,127,128].

### 3.2. Integration of Multi-Omics Data

AI is also a powerful tool for integrating multi-omics data, which is necessary for a more holistic understanding of smoking-induced damage. Conventional approaches face difficulty when interpreting signals across genomics, transcriptomics, imaging, and clinical layers, highlighting the need for models that can discern results from fragmented data [129,130]. Transformer neural networks and advanced DL models are adept at synthesizing multimodal data such as imaging with pathology or genomics data [26,131]. These networks may be valuable for linking persistent molecular alterations with early neoplastic changes observed in histopathologic or radiologic images. Research has shown that AI-driven integration of multi-omics data can refine molecular subtypes, predict prognosis, and identify therapeutic responses in lung cancer [67,124,132]. A key advantage of these models is their ability to identify biomarkers as shifts in coordinated omics patterns across multiple layers, rather than changes in a few isolated genes [65,123]. These integrative models may help reveal how persistent epigenetic marks, such as DNA methylation at AHRR and F2RL3 loci, interact with transcriptomic changes to sustain a pro-tumorigenic microenvironment long after an individual quits smoking [21,38]. 

Traditional bisulfite sequencing remains a gold standard for DNA methylation analysis but involves DNA degradation and limited ability to discriminate between different epigenetic modifications [133,134]. The recent availability of 5-base sequencing technologies, such as PacBio HiFi, Oxford Nanopore duplex, and Illumina’s 5-Base Solution further strengthens these integrative approaches [77,135,136]. Long-read platforms enable the direct detection of multiple base modifications (e.g., 5mC, 5hmC) at single-molecule resolution, while Illumina’s short-read approach presents a parallel method for simultaneous detection of genomic variants and cytosine methylation in one workflow [77,135,136]. Incorporating this additional layer of epigenetic information into multi-omics frameworks enhances the ability of AI-driven models to identify biomarkers as coordinated omics patterns, providing a more comprehensive view of persistent versus nonpersistent smoking-induced alterations.

### 3.3. Acceleration of Biomarker Development and “Virtual Biopsies”

AI can also be used to accelerate the development of biomarkers for risk stratification and chemoprevention [27,137,138]. By analyzing longitudinal molecular and imaging data from former smokers, ML models may identify molecular signatures associated with long-term cancer risk [26,137]. For instance, a DL model could be trained on sequential multi-omics and imaging profiles to predict which individuals are likely to follow a persistence-prone molecular trajectory and would therefore benefit from targeted chemopreventive interventions [27,138]. 

**Figure 3:**
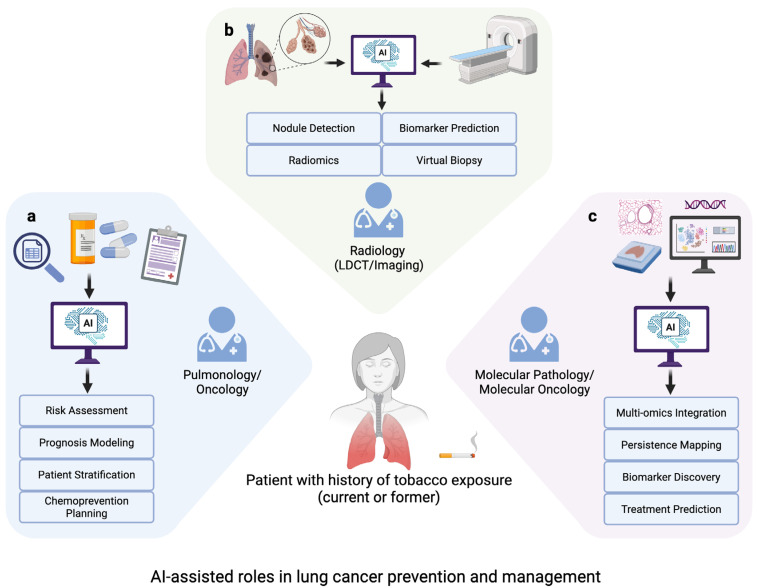
AI-assisted roles in lung cancer prevention and management. A patient with a history of tobacco use can be evaluated across several disciplines with the use of AI. (**a**) For individuals at risk, pulmonologists and oncologists can integrate smoking history, comorbidities, and molecular profiles with AI to predict long-term risk, stratify susceptibility, and guide personalized prevention strategies. (**b**) For screening and early detection, radiologists can apply AI to LDCT and PET imaging for automated nodule detection, segmentation, and radiomic analysis, enabling prediction of persistent structural patterns and non-invasive “virtual biopsies.” (**c**) For confirmed lung cancer cases, molecular pathologists can employ AI to integrate multi-omics, single-cell, and spatial data to distinguish persistent from reversible (persistence mapping) smoking-related changes. These approaches also support the prediction of treatment-relevant biomarkers that may inform future precision-oncology or chemoprevention strategies, potentially accelerating early detection and prevention efforts.

These advanced AI-based models can non-invasively predict molecular features from routine clinical data or images, providing a “virtual biopsy” that may be used to monitor molecular changes over time [26,139]. Virtual biopsies typically involve training AI models on paired radiologic and molecular data; once trained, these models can predict molecular alterations solely from radiological or histopathological images [139,140,141,142]. In practice, these models use paired datasets where imaging-derived features are matched to molecular readouts (e.g., mutation status or expression-related biomarkers), enabling prediction of molecular states from images in independent samples [140,141,142]. When used as adjunctive tools rather than replacements for histopathology, such approaches could enhance low-dose computed tomography (LDCT) screening by linking radiographic findings with molecular signatures and potentially reducing the need for unnecessary invasive procedures [81,82]. Previous research has demonstrated that DL models can predict numerous clinically relevant mutations, such as EGFR, STK11, and KRAS, from H&E-stained pathology slides in lung cancer [141]. This function has been extended to radiologic images, where AI models analyze CT scans to predict driver mutations, like EGFR, and the expression of immunotherapy biomarkers, such as PD-L1 [142,143]. Beane et al. demonstrated the potential for identifying transcriptomic biomarkers by developing a highly accurate classifier from 28 persistently dysregulated genes that could classify former and current smokers [23]. These findings indicate that airway gene-expression patterns may serve as sensitive indicators of prior exposure and long-term risk, and that AI-based models could refine such signatures to distinguish transient from enduring molecular damage. However, the clinical deployment of these approaches requires careful validation and should be viewed as complementary to established diagnostic standards. Together, these applications demonstrate how AI could enhance lung cancer patient care across clinical, imaging, and molecular domains. This multidisciplinary framework is summarized in Figure 3, illustrating AI uses for (a) risk prediction in smokers without cancer, (b) radiologic assessment and virtual biopsies, and (c) mapping persistent molecular alterations in diagnostic or surveillance contexts.

## 4. Limitations, Generalizability, and Future Directions

Despite rapid technological advancements, translating AI-based multi-omics approaches for smoking-related persistence into clinical applications remains limited by biological heterogeneity, data availability, imperfect persistence labels, and methodological constraints [26,28,70]. This section summarizes the primary sources of bias and uncertainty that affect generalizability, interpretability, and clinical relevance, and outlines priorities for future research. 

### 4.1. Cohort Heterogeneity and Generalizability 

Most datasets used to assess smoking-related molecular persistence are ancestry- and geography-biased, and baseline methylation or expression profiles differ across populations, limiting model portability [37,41,70]. Differences in smoking intensity, cumulative exposure, and time since cessation also contribute to heterogeneity and may shift molecular persistence trajectories in ways that are inconsistently captured across studies [8,37]. In addition, biospecimen type strongly influences the biology being measured; persistence signatures derived from blood, airway brushings, or lung tissue may reflect distinct cellular processes and may not replicate across sample types [41,70]. Genetic background can also affect smoking-associated epigenetic responses, introducing inter-individual variability that may challenge transferability when AI models are trained on a single cohort [41]. To improve generalizability, AI models should be developed on sufficiently large and diverse cohorts and evaluated in independent populations. The use of publicly available resources and reproducible workflows is also critical so that findings can be validated across studies [26,28,70].

### 4.2. Interpretation, Causality, and Clinical Relevance 

A key limitation of current persistence frameworks is that most proposed molecular signatures remain observational, and strong statistical associations do not establish whether a marker is mechanistically involved in carcinogenesis or reflects long-lasting exposure history [8,26]. This limitation is especially relevant for persistent epigenetic markers and composite AI-derived signatures that integrate numerous correlated features [26,70]. As a result, AI-based approaches should be framed primarily as tools for risk stratification and hypothesis generation, with causal claims requiring independent functional validation [26,28]. Interpretability of AI models should also be prioritized to link predictions back to underlying biology and clinical context, especially in prevention and early-detection settings [28,139]. Clinical use cases should also be defined conservatively, as histopathology remains the diagnostic gold standard. Virtual biopsy approaches are best positioned as adjunctive decision-support tools rather than replacements, particularly in screening contexts where false positives and downstream harms are clinically meaningful [26,28].

### 4.3. Technical and Methodological Constraints of AI Models

Many AI models applied to lung cancer genomics are underpowered, lack external validation, and rely on performance metrics that may not directly translate to clinical decision-making [26,28,139]. These challenges are amplified in research of smoking-related molecular persistence, as labels often depend on longitudinal follow-up, reducing effective sample sizes and increasing missingness across omics layers [70]. Managing missing data points remains a major technical challenge, since variable assay availability and quality control issues across multi-omics datasets can degrade model performance. Using robust missing data handling techniques, such as imputation or model architectures resilient to missingness, is essential to maintain accuracy and generalizability of AI models [70]. In addition, persistent smoking-associated molecular alterations may arise from clonal expansion of long-lived altered cell populations, so “ground truth” labels often reflect complex mixtures of cell states instead of discrete molecular categories. This biological complexity may reduce model stability when training data are weakly labeled or heterogeneous [55].

### 4.4. Future Directions

Future progress will require larger, more diverse longitudinal cohorts, standardized operational definitions of molecular persistence, integrations with functional and experimental validation, and prospective evaluation prior to clinical integration [8,26,28,70]. Longitudinal sampling is particularly important for modeling molecular recovery as a dynamic process and for distinguishing transient from persistent smoking-induced alterations [8,23]. When possible, considering genetic susceptibility, exposure history, and tissue context will be necessary for creating generalizable models across populations and biospecimens [37,41]. These priorities have been highlighted in recent reviews, which emphasize the need for explainable AI, robust validation, and cautious clinical framing before adoption [26,28,138]. With these priorities in place, the rapid emergence of multimodal frameworks, explainable architectures, and large collaborative datasets establish a strong foundation for the use of AI tools in advancing prevention, monitoring, and early detection in tobacco-related lung cancers.

## 5. Conclusions

Tobacco exposure causes widespread molecular alterations across the airway and lung tissues. While some of these changes undergo partial or complete recovery after smoking cessation, others last for decades and continue to influence disease risk. Persistent alterations, such as aberrant DNA methylation, impaired DNA repair responses, and immune dysregulation, may help explain the lasting vulnerability of former smokers, while ongoing exposure sustains elevated risk in current smokers. Nonpersistent alterations demonstrate the rapid biological benefits of smoking cessation and identify potential avenues for chemoprevention prior to malignant transformation. 

Recognizing which molecular changes are persistent and nonpersistent is essential for refining early detection, risk stratification, and prevention strategies. AI-based methods that combine multi-omics, radiologic, and pathologic data can identify persistence signatures, accelerate biomarker discoveries, and improve individual risk profiling beyond the capabilities of traditional analyses. However, the clinical translation of these approaches remains constrained by cohort heterogeneity, limited longitudinal validation, and challenges related to causal inference and model interpretability. The evidence synthesized in this review supports that persistent smoking-induced molecular alterations represent a distinct biological state that is not solely captured by smoking status. Clarifying which alterations endure across tissues and over time helps define the limits of current prevention strategies, signifying where improved longitudinal and mechanistic studies are most needed. 

By integrating longitudinal, single-cell, and spatial multi-omics datasets along with environmental, genetic, and immune factors, AI can model recovery as a dynamic process and improve predictions of smoking-related molecular persistence. Addressing these limitations through diverse cohorts, standardized definitions of persistence, and prospective validation will be essential prior to the integration of AI frameworks in routine lung cancer prevention or screening strategies. With continued progress, biological and computational insights can be translated into practical tools that guide screening, surveillance, and chemopreventive strategies, ultimately reducing the burden of tobacco-related lung cancer and improving outcomes for the millions of current and former smokers worldwide.

## Figures and Tables

**Table 1:** Representative datasets, sequencing platforms, AI models, and bioinformatics tools across the lung-cancer prevention and precision-oncology workflow. These resources correspond to the stages illustrated in Figure 2 and exemplify how genomic, imaging, and clinical data can be integrated through AI-driven pipelines for biomarker discovery, risk stratification, and clinical decision support [70,71,72,73,74,75,76,77,78,79,80,81,82,83,84,85,86,87,88,89,90,91,92,93,94,95,96,97,98,99,100,101,102,103,104,105,106,107,108,109,110,111,112,113,114,115,116,117,118,119,120,121,122].

Workflow Step	Datasets, Models, or Platforms	Primary Role
Data Acquisition & Integration	TCGA/Genomic Data Commons	Large-scale multimodal genomic and clinical public datasets [70,71].
	LIDC-IDRI/LUNA16/EMRs	Annotated lung nodule CT datasets and electronic medical records for training and validation [72,73].
	PacBio HiFi, Oxford Nanopore Duplex, Illumina platforms	Long- and short-read sequencing for variant and methylation multi-omic profiling [74,75,76,77].
Preprocessing & QC	GATK, SAMtools	Standard pipelines for variant calling and quality-control analysis [78,79].
	PLINK	Genotype data management and association analysis tool [80].
Prevention & Risk Stratification	Sybil	DL model predicting lung-cancer risk from LDCT [81,82].
	XGBoost, PLCOm2012	Gradient-boosting and statistical models for clinical risk prediction [83,84].
AI/ML Frameworks & Core Methods	DL architectures, Transformer Networks, Graph Neural Networks	Neural-network approaches for pattern recognition and modeling of molecular networks and pathways across multimodal data [85,86,87].
	Radiomics pipelines	Quantitative feature extraction from medical imaging to characterize tumor phenotypes [88].
Multi-Omics Integration & Network Analysis	WGCNA, MOFA+/mixOmics	Co-expression network and multi-omic factor analysis for integrated profiling [89,90,91].
	PANDAOmics	AI-driven commercial platform for drug target discovery integrating multi-omic data [92,93].
Segmentation & Image Processing	U-Net, ConvPath	Automated CT and whole-slide image segmentation with CNNs [94,95].
	3D Slicer, PyRadiomics	Extraction of quantitative radiomic features from segmented regions [96,97].
Diagnosis & Lesion Detection	Lunit INSIGHT CXR	AI-based detection of pulmonary nodules and lesions in chest radiographs [98,99].
	Paige.AI, CLAM, TIAToolbox	AI platforms and frameworks for automated histopathological image analysis [100,101,102].
Biomarker Discovery & Pathway Analysis	QIAGEN IPA, GSEA	Pathway enrichment and functional annotation of gene signatures [103,104].
	clusterProfiler, Cytoscape	Functional enrichment and network visualization of molecular interactions [105,106].
	DeepNovo, PepNet, PepFormer	DL tools for peptide sequencing and neoantigen discovery in immunotherapy [107,108,109,110].
Clinical Decision Support	Tempus Lens, FoundationOne CDx, Caris MI	AI-enabled decision-support platforms integrating genomic and clinical data for treatment selection [111,112,113].
	cBioPortal	Interactive platform for exploring multidimensional cancer-genomics data [114].
Validation & Model Refinement	PrecisionFDA	Regulatory benchmarking and reproducibility testing for genomic pipelines [115].
	IGV (Integrative Genomics Viewer)	Visualization tool for validation of variants and expression patterns [116].
General AI/Development Frameworks	QuPath, MONAI, OpenSlide	Open-source libraries for digital pathology and scalable image analysis [117,118,119].
	scikit-learn, TensorFlow, PyTorch	Core ML libraries for model development and deployment [120,121,122].

## Data Availability

No new data were created or analyzed in this study. Data sharing is not applicable to this article.

## Abbreviations

The following abbreviations are used in this manuscript:

5hmC	5-Hydroxymethylcytosine
5mC	5-Methylcytosine
AI	Artificial Intelligence
AHRR	Aryl Hydrocarbon Receptor Repressor
ALDH	Aldehyde Dehydrogenase
CDKN2A/p16	Cyclin Dependent Kinase Inhibitor 2A
cHCC-CCA	Combined Hepatocellular-Cholangiocarcinoma
CI	Confidence Interval
CNN	Convolutional Neural Network
CpG	Cytosine–Phosphate–Guanine Dinucleotide
CT	Computed Tomography
CYP	Cytochrome P450
DL	Deep Learning
DNA	Deoxyribonucleic Acid
EMR	Electronic Medical Record
EGFR	Epidermal Growth Factor Receptor
EWAS	Epigenome-Wide Association Study
F2RL3	Coagulation Factor II (Thrombin) Receptor-Like 3
FHIT	Fragile Histidine Triad
GATK	Genome Analysis Toolkit
GDC	Genomic Data Commons
GSEA	Gene Set Enrichment Analysis
H&E	Hematoxylin and Eosin
IARC	International Agency for Research on Cancer
IGV	Integrative Genomics Viewer
IPA	Ingenuity Pathway Analysis
KRAS	Kirsten Rat Sarcoma Viral Oncogene Homolog
LDCT	Low-Dose Computed Tomography
LIDC-IDRI	Lung Image Database Consortium – Image Database Resource Initiative
LOH	Loss of Heterozygosity
LUNA16	Lung Nodule Analysis 2016 Dataset
LUAD	Lung Adenocarcinoma
LUSC	Lung Squamous Cell Carcinoma
MCCS	Melbourne Collaborative Cohort Study
miRNA	MicroRNA
ML	Machine Learning
MOFA+	Multi-Omics Factor Analysis Plus
MONAI	Medical Open Network for Artificial Intelligence
ncRNA	Noncoding RNA
NNK	4-(Methylnitrosamino)-1-(3-pyridyl)-1-butanone
NOWAC	Norwegian Women and Cancer Study
NSCLC	Non-Small-Cell Lung Cancer
NSHDS	Northern Sweden Health and Disease Study
OR	Odds Ratio
PAH	Polycyclic Aromatic Hydrocarbon
PD-L1	Programmed Death-Ligand 1
PET	Positron Emission Tomography
PI3K	Phosphoinositide 3-Kinase
PLCOm2012	Prostate, Lung, Colorectal, and Ovarian Cancer Screening Trial 2012 Risk Prediction Model
QC	Quality Control
RF	Random Forest
RNA	Ribonucleic Acid
SCLC	Small-Cell Lung Cancer
SVM	Support Vector Machine
TCGA	The Cancer Genome Atlas
TP53	Tumor Protein p53
U-Net	Convolutional Neural Network Architecture for Biomedical Image Segmentation
WGCNA	Weighted Gene Co-expression Network Analysis
XGBoost	eXtreme Gradient Boosting
